# Modeling Linguistic (A)Synchrony: A Case Study of Therapist–Client Interaction

**DOI:** 10.3389/fpsyg.2022.903227

**Published:** 2022-05-23

**Authors:** Dennis Tay, Han Qiu

**Affiliations:** Department of English and Communication, The Hong Kong Polytechnic University, Kowloon, Hong Kong SAR, China

**Keywords:** linguistic synchrony, synchrony measure, LIWC, cluster analysis, psychotherapy talk

## Abstract

Interpersonal synchrony is the alignment of responses between social interactants, and is linked to positive outcomes including cooperative behavior, affiliation, and compassion in different social contexts. Language is noted as a key aspect of interpersonal synchrony, but different strands of existing work on linguistic (a)synchrony tends to be methodologically polarized. We introduce a more complementary approach to model linguistic (a)synchrony that is applicable across different interactional contexts, using psychotherapy talk as a case study. We define linguistic synchrony as similarity between linguistic choices that reflect therapists and clients’ socio-psychological stances. Our approach involves (i) computing linguistic variables per session, (ii) k-means cluster analysis to derive a global synchrony measure per dyad, and (iii) qualitative analysis of sample extracts from each dyad. This is demonstrated on sample dyads from psychoanalysis, cognitive-behavioral, and humanistic therapy. The resulting synchrony measures reflect the general philosophy of these therapy types, while further qualitative analyses reveal how (a)synchrony is contextually co-constructed. Our approach provides a systematic and replicable tool for research and self-reflection in psychotherapy and other types of purposive dialogic interaction, on more representative and limited datasets alike.

## Introduction

Interpersonal synchrony is the alignment of neural, perceptual, affective, physiological, and behavioral responses during social interaction (Semin and Cacioppo, 2008). Examples include synchronous breathing patterns, movement, gesture, and language use. These are linked to positive evaluations of teacher–student (Bernieri, 1988) and spousal relationships (Julien et al., 2000), and are claimed to promote cooperative behavior, affiliation, and compassion (Hove and Risen, 2009; Valdesolo and DeSteno, 2011). Psychotherapy is a highly relevant context for investigating interpersonal synchrony, as therapists assist clients to modify behaviors, cognitions, and emotions through verbal interaction (Norcross, 1990). Koole and Tschacher (2016) outline three interlinked levels of synchronous processes: perceptual-motor processes like movement, facial expressions, and gestures, complex cognitive processes like memory and language, and emotion regulation. These are claimed to strengthen the therapeutic alliance between therapists and clients, leading to more positive treatment outcomes (Ardito and Rabellino, 2011).

The key role of language in psychotherapy has fostered considerable interest in the nature of linguistic synchrony in therapist–client interaction. It is generally thought that therapists and clients attain synchrony by developing shared mental representations, or a “common language,” through “mutual adaptation to another’s linguistic behaviors” (Koole and Tschacher, 2016, p. 7). However, existing approaches to linguistic synchrony tend to be polarized along the usual qualitative–quantitative divide, focusing on disparate levels and units of analysis. The aim of this paper is to demonstrate a more systematic and integrated approach that draws from different methods to model linguistic (a)synchrony at the sessional level across the treatment span, using fairly accessible tools and techniques. Synchrony is defined as the extent of similarity between linguistic choices of therapists and clients that reflect socio-psychological stances adopted in interaction. Our approach consists of the following steps: (i) automated quantification of key therapist and client language variables on a session-by-session basis, (ii) cluster analysis to identify (dis)similar sessions as (a)synchronized, and (iii) qualitative analysis of examples in context to illustrate the varied co-construction of (a)synchrony. We demonstrate these steps with a case study of three sample dyads from different psychotherapy approaches—psychoanalysis, cognitive-behavioral, and humanistic therapy. The main aim is to offer a systematic and replicable method for basic linguistic (a)synchrony research, which could be applied to more representative datasets as well as other social contexts of a similar interactive nature like classrooms and online fora. Interested practitioners could also apply the approach to understand and reflect on their own (a)synchronous language use with clients. We begin by reviewing existing theoretical and methodological approaches to linguistic synchrony, their applications to psychotherapy, and the limitations that motivate the present proposal. We then demonstrate each step of our approach and discuss the attendant implications. Directions for future research are offered in the end.

## Approaches to Linguistic Synchrony

The influential Interactive Alignment Model (Pickering and Garrod, 2004) synthesizes previous related work (Sacks et al., 1974; Brennan and Clark, 1996; Zwaan and Radvansky, 1998) to provide a cognitive account of linguistic synchrony. The main idea is that speakers in natural dialogue prime each other to develop aligned representations across phonological, syntactic, semantic, and situational levels. Each level has its own monitoring and repair mechanisms, and alignment at one level reinforces other levels to enhance the overall perception of synchrony. Since this process is assumed to be primitive and unconscious, the model is less able to account for complexities like higher-order communication strategies, deliberate attempts to (mis)align with each other, and other context-specific features emergent in spontaneous interaction. Psychotherapy is seldom discussed in this model but is a case where we would precisely expect to see such complexities (Anderson and Goolishian, 1988). Elsewhere, communication and language researchers consider these complexities to be of primary interest. Communication Accommodation Theory (Giles, 2016), for example, claims that our interactions are consciously motivated by their perceived social consequences. People linguistically align/synchronize with one another, or vice versa, to reduce or accentuate differences as desired. This has been demonstrated in linguistic analyses of various social scenarios like intercultural language education (Dörnyei and Csizér, 2005), law enforcement (Giles et al., 2006), and in fact psychotherapy (Ferrara, 1991, 1994). Ferrara (1994, p. 5) notes that therapists and clients use the core strategies of “repetition” and “contiguity” to construct meaning in accommodative ways, “taking up portions of the other’s speech to interweave with their own.” Another leading approach to psychotherapy talk is conversation analysis, which focuses on the turn-by-turn architecture of natural dialogue and has been usefully applied to examine therapist–client language both within and across different therapy approaches (Peräkylä et al., 2011; Weiste and Peräkylä, 2013). The main idea is that therapeutic processes including synchrony are achieved by patterns of interaction manifested in sequential conversational structures. This notion of collaborative linguistic alignment cuts across other related research including the act of “wordsmithing” in counseling (Strong, 2006), the strategic communication of risks (Sarangi et al., 2003), and the co-construction of figurative language by therapists and clients (Kopp, 1995; Tay, 2016, 2021a). We can observe in all of the above work a preference for nuanced qualitative analysis of “localized” linguistic units, or “isolated snapshots” (Brown et al., 1996), like a single conversational turn or topic. An inevitable trade-off is the inability to depict (a)synchrony at higher and perhaps more natural levels of organization. A prime example is the institutionalized level of the session itself. For both therapists and clients, sessions are likely to be more intuitive, concretely experienced, and recallable than single turns or topics. Despite this, there is little work on how language manifests (a)synchrony at sessional level, in large part because it is hard to analyze entire sessions in a nuanced yet reliable way. Complementary quantitative methods are required for this.

Computational linguistics research, which applies computational techniques to analyze natural language, offers potential quantitative solutions for evaluating (a)synchrony. On the unconscious-versus-strategic alignment debate described above, computational evidence suggests that “alignment is not a completely automatic process but rather one of many discourse strategies that speakers use to achieve their conversational goals” (Doyle and Frank, 2016). This is an invitation to explore contexts like psychotherapy where such conversational goals are relatively well-defined. Furthermore, due to the relative concreteness of words over other grammatical levels (Gries, 2005; Healey et al., 2014), there is a general preference for quantification at word level for the English language. Relevant research has yielded two types of synchrony measures: distributional and conditional. Distributional measures include the Zelig Quotient (Jones et al., 2014) and Linguistic Style Matching (Niederhoffer and Pennebaker, 2002), which determine linguistic (dis)similarity and/or correlation between independent units of analysis. Conditional measures like Local Linguistic Alignment (Fusaroli et al., 2012) focus instead on the relationship between adjacent units—somewhat in the vein of conversation analysis described above. Both types are complementary because distributional measures show global similarity but not necessarily the contextual qualities of alignment, and vice versa for conditional measures. However, they are seldom applied together in order to deepen understanding of synchrony in a specific context like psychotherapy. Also noteworthy is that recent work tends to emphasize the importance of function or grammatical words. This is because while content words are often tied to arbitrarily changing topics, grammatical words are more context-invariant and thus more revealing of speakers’ interactional styles (Doyle and Frank, 2016). This is the intuition behind the presently used automated text analytic software like Linguistic Inquiry and Word Count (LIWC; Tausczik and Pennebaker, 2010), which relies heavily on grammatical categories to score texts (see below). Quantitative analyses of these scores with respect to sessional progress could then reveal patterns to be further investigated with qualitative examples in context.

In summary, linguistic synchrony research in contexts like psychotherapy would benefit from a more explicit sessional focus and complementary techniques to model and interpret both the global and contextual aspects of (a)synchrony. The following method section will detail each step of the presently proposed approach, and the results and discussion section will report a demonstration of the approach on three sample dyads from different therapy types.

## Materials and Methods

### Step 1: Quantification of Therapist and Client Language With LIWC

LIWC is a computer text analysis program used in many language and discourse studies. Its motivating assumption is that “the words we use in daily life reflect what we are paying attention to, what we are thinking about, what we are trying to avoid, how we are feeling, and how we are organizing and analyzing our worlds” (Tausczik and Pennebaker, 2010, p. 30)—in short, various socio-psychological stances we adopt when communicating. Given an input text, LIWC computes the frequencies of a large number of content and grammatical word categories defined by a built-in dictionary. It can further compute four “summary variables” as combinations of the above categories. These are called *analytical thinking, clout, authenticity,* and *emotional tone*, and are the focus of the present approach. Table 1 shows the summary variables, the categories that define them, and corresponding studies that show how they reliably differentiate input texts. The plus signs (+) indicate categories that are relatively frequent in texts that reflect a higher level of that summary variable, and vice versa for minus signs (−). Detailed psychometric properties of the latest version (LIWC-22) are found in Boyd et al. (2022).

**Table 1:** LIWC summary variables.

Summary variable	Defining grammatical and content word categories
Analytical thinking	+articles, prepositions –pronouns, auxiliary verbs, conjunctions, adverbs, negations (Pennebaker et al., 2014)
Clout	+1st person plural pronouns, 2nd person pronouns –tentative words (e.g., *maybe* and *perhaps*; Kacewicz et al., 2013)
Authenticity	+1st person singular pronouns, 3rd person pronouns, exclusive words (e.g., *but, except, without*) –negative emotion words (e.g., *hurt, ugly,* and *nasty*), motion verbs (e.g., *walk, move*, and *go*; Newman et al., 2003)
Emotional tone	+positive emotion words (e.g., *love, nice*, and *sweet*) –negative emotion words (e.g., *hurt, ugly,* and *nasty*; Cohn et al., 2004)

Each summary variable for an input text is given a standardized score of 0–100. A high *analytical thinking* score reflects formal, logical, and hierarchical thinking versus informal, personal, here-and-now, and narrative thinking. This is based on college admission essays where those with more articles and prepositions were rated as more formal and precise in describing objects, events, goals, and plans, while those with more pronouns, auxiliary verbs etc. involved more personal stories (Pennebaker et al., 2014). A high *clout* score reflects expertise and confidence versus tentativeness and humility. This is based on decision-making tasks, chats, and personal correspondences. Higher status individuals used more *we/our, you/your* and fewer tentative words as they tend to be more other-focused and less unsure than lower status individuals (Kacewicz et al., 2013). A high *authenticity* score reflects more honest, personal, and disclosing, versus more guarded and distanced language. This is based on elicited true and false stories where the latter has fewer first and third person pronouns, exclusive words, and more negative emotion and motion verbs. One explanation is that liars tend to dissociate themselves with the lie, feel more tension and guilt, and speak in less complex ways (Newman et al., 2003). Lastly, a high *emotional tone* score suggests a more positive and upbeat style, a low score anxiety/sadness/hostility, and a score around 50 a lack of emotionality. This is based on diaries around September 11, 2001 where negative emotion words increased sharply following the attack and gradually returned to pre-attack baselines after some time (Cohn et al., 2004).

Recent studies (Huston et al., 2019; Tay, 2020; Qiu and Tay, 2022) highlight the potential for the four summary variables to profile how language is used in therapeutic work. They reflect aspects like how narratives are told, the stance of therapists when dispensing advice and of clients when receiving it, the negotiation of relationships, and linguistic displays of emotional states. For example, therapists could speak in a highly logical way (*analytic thinking*), but hedge their advice (*clout*) and use more positive words (*emotional tone*) to come across as more personal (*authenticity*) and optimistic. Similarly, other studies have applied LIWC to profile how language works across diverse contexts like social media (Tay, 2021b) and news (Smirnova et al., 2017). By splitting therapist and client language in each session transcript, we derive a multivariate linguistic profile on a per-session basis across the treatment span. The extent to which a dyad is synchronized could thus be defined in terms of how similar the therapist and client variable scores are within and across sessions, as measured by cluster analysis in “Step 2”. While LIWC is featured in the present demonstration, it should be noted that in principle, this multivariate linguistic profile can also be derived using other coding or quantification schemes such as expert ratings and different text analysis programmes.

### Step 2: Cluster Analysis of Sessions

Cluster analysis is the task of grouping a set of objects based on their properties, such that those in the same group (or cluster) are maximally similar and each group is maximally dissimilar to one another. It is used in classification tasks in diverse fields like marketing, image analysis, and bioinformatics, on quantitative as well as qualitative data types (Guest and McLellan, 2003).

There are many clustering algorithms that differ on key parameters like how (dis)similarity is defined and what constitutes a cluster (Yim and Ramdeen, 2015). Most generally, clustering algorithms are hierarchical or non-hierarchical, and the choice of algorithm depends on the objectives and data at hand. Hierarchical algorithms operate in a top-to-bottom manner such that smaller clusters are part of larger ones, leaving the analyst to decide how many clusters should be interpreted. The most common example is agglomerative hierarchical clustering. Non-hierarchical algorithms, on the other hand, aims to optimize some overall evaluation criterion, and in so doing allows the analyst to investigate the optimal number of clusters. The most common example is *k*-means clustering (where *k* = no. of clusters). Cluster analysis can be performed on most statistical software packages. We use the *Python* 3.8 programming language, code available upon request.

For the present application, the set of objects to be clustered are the session transcripts of therapist-only and client-only language. For example, a 10-session dyad will have 20 objects labeled T_1_–T_10_ and C_1_–C_10_. We call these “sub-transcripts.” The properties of each sub-transcript are its LIWC variable scores computed in “Step 1.” Whichever algorithm is preferred, the aim is to offer a distributional measure of synchrony where for each session *x*, if sub-transcripts T*_x_* and C*_x_* fall into the same cluster, then session *x* is considered synchronized. Otherwise, session *x* is asynchronized. The basic rationale is that therapist and client language within the same session ought to be more similar to each other than they are to other sessions, if we want to claim that session as synchronized. Step 2, therefore, yields the following concrete outcomes: (i) which sessions across the treatment span are (a)synchronized, (ii) the percentage of synchronized vs. asynchronized sessions as an overall measure of the dyad, and (iii) the distribution pattern of (a)synchrony across time. Each outcome can be further probed with reference to the actual transcripts in context.

### Step 3: Qualitative Analysis in Context

Qualitative analysis of examples in context is integral to virtually all linguistic approaches to psychotherapy. This may be complemented by relevant quantitative analysis either before, after, or in an intermittent way (Creswell, 2014). A key rationale for performing quantitative prior to qualitative analysis like in the present case is that the former provides motivated entry points and/or criteria for elaborating specific examples in more focused and context-sensitive ways (Tay, 2019). In our case, the Step 2 synchrony measures will be further qualitatively discussed at two levels. At the general level of therapy type, we query the extent to which each dyad’s synchrony measure reflects the philosophy of its therapy type. While we should not expect any particular dyad to “perfectly” enact its underpinning theoretical principles, this level of analysis is helpful as a backdrop for larger comparisons of linguistic (a)synchrony across types, as well as individual practitioners’ self-reflection on their own theoretical (mis)alignments. We then move to the specific level of examining *how* and *why* (a)synchrony is co-constructed in selected extracts. As mentioned earlier, this helps us connect the “global” quantitative synchrony measures with what actually goes on in therapist–client interaction, and potentially reveals higher-order communication strategies as well as other context-specific features that may deepen our understanding of the nature of (a)synchrony.

## Results and Discussion

### Sample Dyads and Demographics

We now demonstrate the above steps on three sample dyads from different therapy types. The source of the transcripts is *Counseling and Psychotherapy Transcripts,* an online subscription database for research and education.^1^ The publishers state that they “adhere to the American Psychological Association’s Ethics Guidelines for use and anonymity, so users can rely on the information for its accuracy and diversity.” Dyad A (15 sessions) is selected from the psychoanalysis therapy type, Dyad B (14 sessions) from cognitive-behavioral therapy, and Dyad C (20 sessions) from humanistic therapy. The three dyads are selected in order to maximize comparability. The clients share broadly similar demographics and presenting conditions: all three are heterosexual white American females in their early- to late-20s diagnosed with anxiety disorder and depression. They all report relationship issues with their parents/spouse/partner. Nevertheless, given the unique nature of each dyad, we must emphasize that they can only illustrate but not represent the therapy types. This again underlines the case study-oriented nature of our approach—it could be applied to larger datasets to make stronger claims about therapy types if desired, as well as more limited ones for purposes like practitioner self-reflection.

### Step 1

Table 2 shows the outcome of Step 1 with the four LIWC summary variable scores for each sub-transcript.

**Table 2:** Summary variable scores for the three dyads.

Dyad A (Psychoanalysis)					Dyad B (CBT)				
**Session**	**Analytic**	**Clout**	**Authentic**	**Tone**	**Session**	**Analytic**	**Clout**	**Authentic**	**Tone**
C1	12.62	41.26	63.11	67.58	C1	7.2	11.6	95.16	59.63
C2	17.73	21.95	69.21	60.4	C2	4.59	14.94	92.75	60.95
C3	35.24	20.39	80.5	66.26	C3	8.49	15.99	92.94	51.18
C4	30.8	25.32	68.91	40.86	C4	6.76	16.41	88.04	50.45
C5	10.08	14.08	69.39	66.7	C5	5.26	19.92	83.7	81.93
C6	19.74	35.59	69.84	50.09	C6	4.23	38.41	73.35	34.36
C7	21.73	42.54	72.59	49.84	C7	5.38	9.82	92.72	41.34
C8	15.09	51.7	28.18	42.92	C8	1.89	8.74	97.23	48.31
C9	20.46	14.71	87.51	49.69	C9	5.35	20.47	92.83	50.54
C10	10.43	32.1	67.85	50.33	C10	3.22	20.56	85.43	44.17
C11	17.74	32.18	67.41	77.29	C11	5.08	8.66	95.22	75.85
C12	11.08	18.31	86.55	67.23	C12	6.09	16.98	86.3	43.6
C13	17.27	25.92	56.74	75.06	C13	5.75	32.15	76.13	64.51
C14	21.27	15.99	87.01	56.85	C14	6.27	18.84	85.88	78.6
C15	29.99	37.22	52.08	41.54	T1	15.4	90.23	50.24	58.7
T1	33.45	73.58	72.27	43.14	T2	11.88	92.89	51.82	62.78
T2	7.39	69.04	69	72.32	T3	17.9	88.02	54.4	64.12
T3	9.49	60.35	66.71	42.41	T4	16.87	93.37	46.92	61.21
T4	20.15	65.65	50.19	22.31	T5	9.17	94.7	47.28	74.81
T5	21.73	61.72	50.61	58.63	T6	15.01	91.05	44.97	74.45
T6	18.49	55.65	59.94	30.12	T7	11.37	87.56	59.66	46.79
T7	20.23	71.69	56.41	31.35	T8	12.74	90.6	57.11	72.19
T8	11.39	63.4	24.98	28.62	T9	17.91	88.03	40.12	51.58
T9	30.32	67.62	67.4	68.9	T10	12.06	90.37	39.1	58.91
T10	21.96	34.07	73.84	49.06	T11	19.2	92.56	56.03	75.48
T11	15.17	48.14	65	85.53	T12	16.37	92.4	48.83	74.9
T12	13.79	68.94	86.4	91.04	T13	15.69	80.49	60.4	65.98
T13	19.69	64.82	75.53	44.51	T14	24.48	87.23	43.96	72.94
T14	38.33	80.23	73.1	91.52					
T15	37.64	75.49	54.67	56.41					
**Dyad C (Humanistic)**									
**Session**	**Analytic**	**Clout**	**Authentic**	**Tone**	**Session**	**Analytic**	**Clout**	**Authentic**	**Tone**
C1	8.31	20.79	83.95	30.48	T1	9.33	84.32	53.22	45.87
C2	9.76	17.3	84.89	39.14	T2	10.82	71.38	50.59	41.91
C3	12.23	34.34	77.4	37.81	T3	14.26	60.8	59.47	34.18
C4	5.99	12.05	89.67	33.92	T4	3.63	46.31	68.51	46.11
C5	7.8	23.77	84.25	52.62	T5	4.78	68.84	55.12	30.38
C6	6.86	15.47	84.35	28.09	T6	5.17	69.12	70.92	35.67
C7	10.71	14.55	84.31	42.98	T7	8.82	50.61	76.79	42.31
C8	13.8	16.09	89.81	56.77	T8	20.29	67.18	66.65	50.7
C9	7.4	19	80.6	33.32	T9	5.65	47.84	78.43	43.22
C10	8.4	20.27	76.16	27.55	T10	9.7	73.77	66.41	37.37
C11	12.11	12.48	84.71	64.38	T11	2.54	43.82	85.18	51.45
C12	9	13.76	89.37	35.98	T12	7.23	45.65	84.16	30.59
C13	8.1	32.02	73.69	31.32	T13	8.09	62.79	72.3	28.49
C14	10.38	10.52	96.12	36.5	T14	6.27	37.4	86.97	15.06
C15	9.93	24.98	89.52	34.03	T15	20.86	70.2	51.28	28.22
C16	16.14	22.22	85.27	40.3	T16	10.81	56.49	67.51	61.87
C17	13.69	38.62	65.02	28.35	T17	7.25	63.79	75.51	16.18
C18	8.72	21.52	84.27	34.38	T18	8.91	70.43	71.29	50.49
C19	9.96	25.35	90.8	61.12	T19	3.91	91.21	60.97	64.83
C20	10.09	11.12	92.02	41	T20	12.18	46.21	75.23	40.69

### Step 2

The next step is to perform cluster analysis using the LIWC variable scores above. The scores are first pre-processed by converting each to a standardized *z*-score, or number of standard deviations from the variable mean score. This is not critical because all four variables are measured on the same scale of 0–100, but must be done otherwise to prevent scales of larger magnitude from biasing cluster formation. Some researchers also advise performing mean-centering in cases where within-person scores (i.e., therapist or client) are likely to be more consistent than between-person scores (i.e., therapist vs. client), leading to entangled within and between-subject effects. There is debate regarding the benefits of mean-centering (e.g., Olvera Astivia and Kroc, 2019), one of the main points being that it does not always alter clustering outcomes. Mean-centering will not be performed in the present demonstration, but readers may do so by simply subtracting each score by the average score of that variable for that person (therapist or client).

The present choice of algorithm is *k*-means clustering (Kodinariya and Makwana, 2013). As mentioned above, each algorithm has different features with relative (dis)advantages, and readers may consult Rodriguez et al. (2019) for a fuller account beyond the present scope. For an alternative hierarchical clustering approach to psychotherapy talk, readers may refer to Tay (2020).

*K*-means clustering essentially defines each cluster as a “centroid,” or point in space, and assigns each object to its nearest cluster. The objective is to minimize the overall distance (also known as “distortion”) between the clusters and objects. This distance inherently decreases with an increasing number of clusters, reaching zero when the number of clusters equals the number of objects. However, since too many clusters are neither parsimonious nor analytically useful, we determine an optimal number by comparing distortion values for a range of cluster numbers. The resulting “elbow plot” shows how distortion decreases as the number of clusters increase. The optimal point, which looks like an elbow, is the number after which the decrease in distortion tapers off. Introducing more clusters after this point no longer improves distortion at the same rate as before, and hence does not compensate for the loss of parsimony.

**Figure 1:**
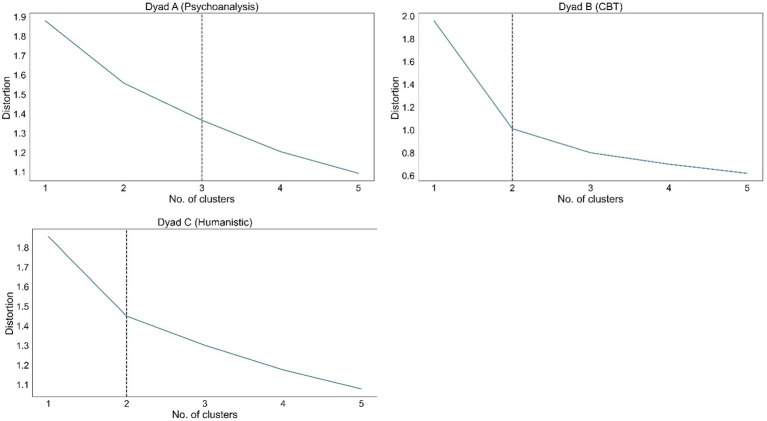
Elbow plots for the three dyads.

Figure 1 shows the elbow plots for Dyads A–C with the vertical lines indicating the elbows. The optimal number of clusters is three, two, and two, respectively.

We then run the *k*-means clustering algorithm for each dyad using the optimal number. The outcome, known as the “clustering solution,” is a unique assignment of each sub-transcript to one of the clusters. This outcome is validated to ensure that the clusters are indeed sufficiently distinct from one another. An efficient validation method is to compare the mean variable scores of members between clusters. These mean scores are also known as “cluster centers.” A good outcome is one where the magnitude and/or direction of these means are clearly distinct, suggesting that the clusters they constitute are also distinct.

**Figure 2:**
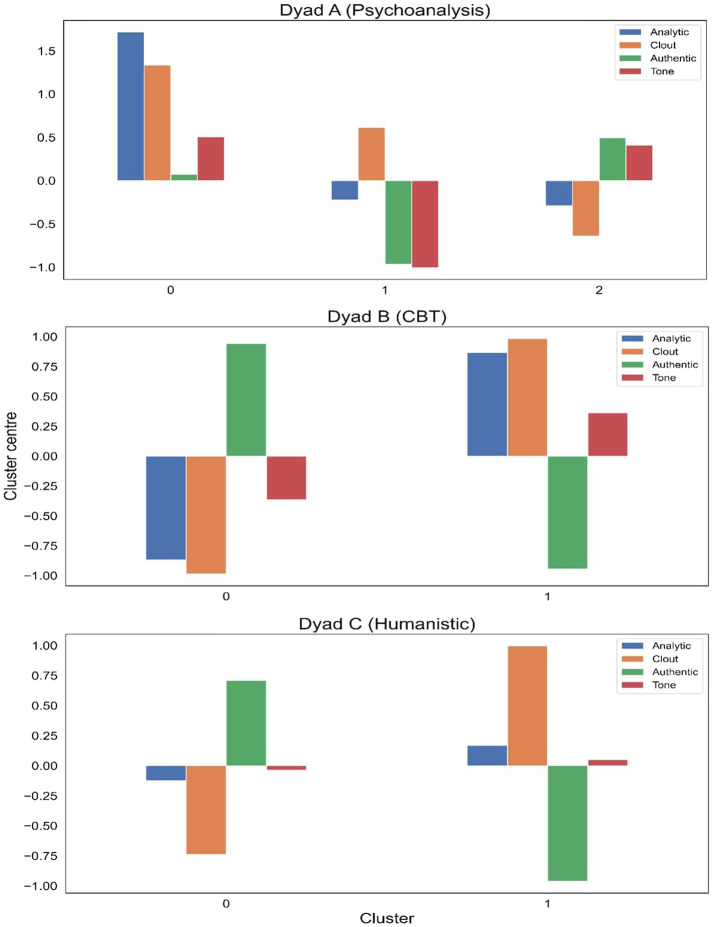
Cluster centers of the three dyads.

Figure 2 plots the cluster centers for each of the three dyads. Two points should be noted: (i) the clusters are “relativized” in the sense that they are computed to delineate as clear a boundary as possible between one another. Therefore, for example, the low *authenticity* and *emotional tone* scores for the second psychoanalysis cluster only mean that they are relatively lower in sub-transcripts in that cluster, compared to the other two psychoanalysis clusters; (ii) recall that the variable scores were converted to *z*-scores, so negative values mean that the scores are below the average of that dyad. Visual inspection adequately shows that the cluster centers are indeed distinct in magnitude and/or direction.

The final step is simply to note which sub-transcripts belong to which clusters in order to determine (i) which sessions across the treatment span are (a)synchronized, (ii) the percentage of synchronized sessions as an overall measure of the dyad, and (iii) the distribution pattern of (a)synchrony across time. Note that the number of (a)synchronized sessions in each dyad might be systematically influenced by the optimal number of clusters, a point to be investigated in future work. Table 3 summarizes these synchrony outcomes with green cells indicating synchrony and red cells asynchrony. Five out of 15 sessions (33.3%) in Dyad A are synchronized, and 5 out of 20 sessions (25%) in Dyad C are synchronized. Remarkably, none of the sessions in Dyad B are synchronized. The distribution across sessions (left to right) provides a visual overview of where in the treatment span synchrony occurs. Salient patterns like contiguous or intermittent blocks could offer additional interpretative insight, as explained later.

**Table 3:** Synchrony outcomes of the three dyads.

Dyad	Synchronized sessions	Synchronized sessions (%)	Synchrony distribution across sessions																			
A	2, 8, 10, 11, 12	33.3	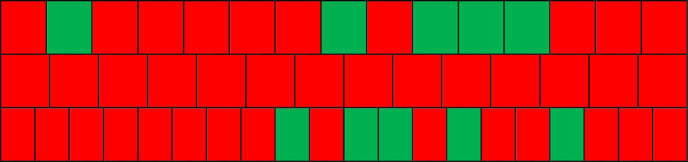
B	–	0	
C	9, 11, 12, 14, 17	25	

**Figure 3:**
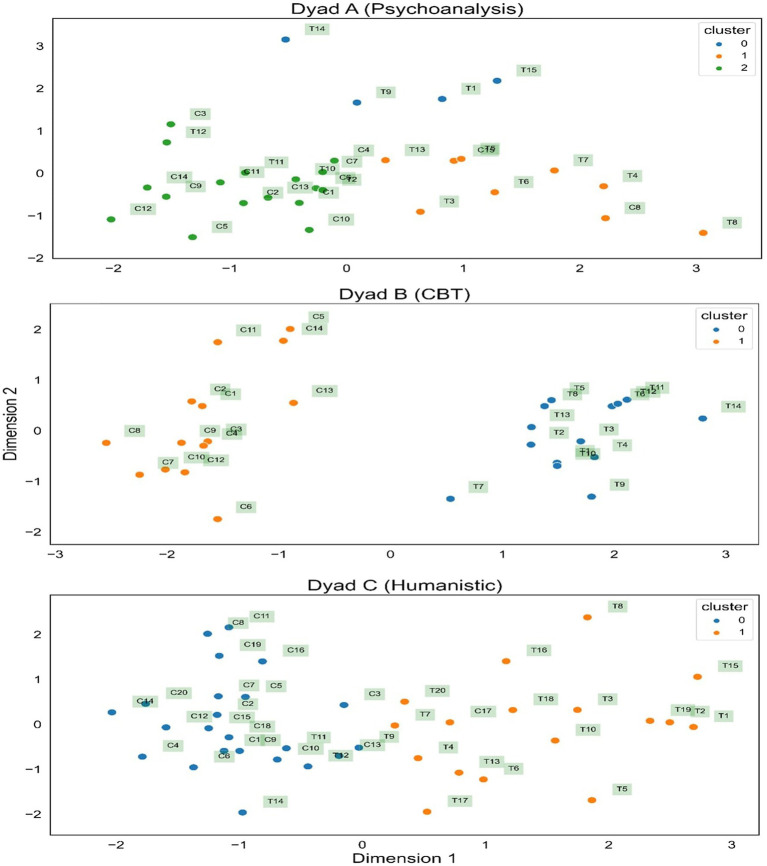
Cluster membership in the three dyads.

As a final visualization, Figure 3 is a spatial representation of each dyad showing how close/similar the sub-transcripts (represented by dots) are. Cluster membership is indicated by color and each sub-transcript is annotated. The location of each sub-transcript is actually in a four-dimensional space because it is defined by the four summary variables. However, since it is impossible to visualize four dimensions, the figure is derived by condensing the four variables into two dimensions using principal components analysis. Despite some inevitable information loss, Figure 3 shows that members of the same cluster are closer together and that Dyad B has a distinct polarization of client and therapist sub-transcripts.

### Step 3

We proceed to the final step of qualitative analysis at the two levels as described above: (i) a discussion of whether the synchrony measures reflect the general philosophy of their represented therapy type, and (ii) a more specific analysis of transcripts in context for how (a)synchrony is interactionally constructed across the three dyads.

Firstly, the measures show that the psychoanalysis sample dyad is the most synchronized (33%), followed by humanistic therapy (25%) and CBT (0%). Psychoanalytic and humanistic interventions are known to be less structured and adherent to established models, attaching more importance to the therapist–client relationship (Arkowitz and Hannah, 1989; Bland and DeRobertis, 2020). Humanistic therapy uses this relation as a primary curative factor and emphasizes attentiveness to the client’s experiential and affective world (Rogers, 1951), while psychoanalysis uses therapist–client interaction as a tool to probe into the client’s repressed thoughts, feelings, and interactional patterns (Kramer et al., 2008). Both these approaches are also less educative in that therapists avoid imposing solutions and interpretations (Watson et al., 2011), and try to “reflect” clients’ unconscious in a neutral manner (Freud, 1924) such that positive changes emerge as a natural result rather than a preconceived goal. Conversely, CBT is an educative, problem-focused, and task-based approach reliant on established techniques (Fenn and Byrne, 2013). Therapists demonstrate their expertise in more explicit ways to “teach” clients to develop more adaptive ways of thinking and behaving. Based on these general philosophical differences, we might speculatively suggest that psychoanalytic and humanistic dyads have greater linguistic synchrony especially when co-constructing shared understandings of clients’ life situations. CBT, on the other hand, is likely to have greater asymmetries in therapist and client linguistic styles.

Our sample dyads generally conform to these expectations. The psychoanalytic (33%) and humanistic dyads (25%) are comparable and both much higher than CBT (0%). It is also possible to examine the distribution of synchronized sessions across time. In our case, the psychoanalytic dyad is barely synchronized at the beginning of treatment but experiences four near-consecutive synchronized sessions near the end, while synchrony in the humanistic dyad is more intermittent, also in the latter half of treatment. The relatively greater display of synchrony in the latter half is likely a result of more co-constructive interaction as treatment develops, with the consecutive block in the psychoanalytic dyad indicating a prolonged period of such activity. Importantly, we are neither claiming that the above synchrony measures correlate with real or perceived treatment quality/outcomes, nor that certain approaches inherently (dis)prefer synchrony because of high/low measures. These are important but ultimately empirical questions that lie beyond the methodological aim of the present study.

In addition to the above cluster analytic insights, a more detailed level of analysis would help researchers and self-reflecting practitioners uncover specific details about the linguistic and interactional construction of (a)synchrony. The present striking example of CBT’s “perfect asynchrony” might be particularly illustrative in this regard. We now demonstrate how qualitative linguistic analysis, key to the various discourse analytic research approaches outlined earlier, can be useful for both research and critical reflection on one’s own practice (Spong, 2010). Recall that whether the dyad is judged to be synchronized in a particular session depends on their holistic similarity across all four LIWC variables. While each word is attributable to and contributes to a specific variable score (Pennebaker et al., 2015), a correspondingly holistic approach when doing qualitative linguistic analysis is to focus on the overall language use in context rather than examining specific word-variable correspondences. Each of the three dyads will therefore be illustrated by one illustrative extract below. Even though the actual words in the extract might not have directly determined their LIWC scores, the aim is to show how both types of analysis cohere and complement each other. As mentioned earlier, all three clients share similar demographics which helps to maximize their comparability. The extracts were accordingly also selected on this basis, in that they all zoom in on a discussion of the client’s difficulties in relating with a specific individual. Furthermore, because the main objective for all three dyads is to resolve these difficulties, it is reasonable to assume that the interactional styles featured in each of these extracts will recur throughout the rest of their respective sessions.

Extract 1 is from dyad A (psychoanalysis) Session 8, a synchronized session (see Table 3). Recall that dyad A has the highest synchrony measure of 33% in the present dataset. The client is relating her boyfriend’s problems at work and her frustration at not being able to help. The therapist is guiding the client to re-experience her emotional disturbances and pin down the cause of her reactions.

1. CLIENT: So he’s worked now for four years in this job, and it’s going to be so hard for him to turn the other way and I cannot will him to do anything.
2. THERAPIST: Yeah. I guess I’m imagining that, seeing him suffer this way and be himself so sort of helpless and being so helpless yourself to do too much about it, is part of what makes it so difficult.
3. CLIENT: Um hmm. So I sense some urgency in the, like, speeding up, or in getting the most out of therapy while he has it. That is my getting-the-most-out-of-things tendency. He does not feel this way. He′s like, “Ah, she just told me I was punishing myself.”
4. THERAPIST: Hmm.
5. CLIENT: Like, yeah. That’s the point.
6. THERAPIST: It’s pretty hard to sit by, huh?
7. CLIENT: Yeah. It’s so hard. It was so much harder in college though. God, I was like, um, I felt that I could not go on in the relationship a number of times.
8. THERAPIST: I mean I guess the place to look would be you know, um, I mean, it does almost like you have vicariously experienced his stress and, except that you are helpless cause you cannot do all the things that you would have done if it were you. But it was him.
9. CLIENT: Yeah.
10. THERAPIST: You know, that is what I imagine used to hang you up about this.
11. CLIENT: Yeah, that was probably the main thing.

Referring back to Table 2, in this session the therapist and client have very similar scores for *analytical thinking* (Client = 15.09, Therapist = 11.39), *clout* (Client = 51.7, Therapist = 63.4), and *authenticity* (Client = 28.18, Therapist = 24.97), with a larger difference for *emotional tone* (Client = 42.92, Therapist = 28.62). These similarities, which constitute the basis for the statistical determination of synchrony, are reflected at a more general level by the observable level of concord between therapist and client. Markers of agreement like “yeah” (Turn 2, 5, 7, 9, and 11) and “um hmm” (Turn 3) suggest that the dyad are gradually co-constructing a shared interpretation of the client’s situation. Their similarly low *analytical thinking* indicates a mutually informal, personal, here-and-now, and narrative style, as the therapist guides the client to explore the underlying meanings, causes, and mechanisms of her thoughts and feelings. The therapist’s display of mid-level *clout* is noticeable. On the one hand, she asserts her interpretations by frequently using ‘I’ and directs them towards the client with “you” (turns 2, 8, and 10). On the other hand, she carefully reduces the force of these interpretations with hedging expressions like “I guess” (Turn 2), “almost like” (Turn 8), and “I imagine” (Turn 10). The client in turn does not display a significantly lower clout as she appeared to respond well to this approach, concurring with the therapist’s interpretations. These observations also account for the similar relatively low levels of *authenticity*—unsurprisingly, given the general psychoanalytic aim of “making the implicit explicit,” clients may find themselves speaking in a more guarded and distanced manner when working through repressed thoughts and feelings. More interesting is the observation that the therapist also displays a comparable level of authenticity, which may suggest an explicit effort to ‘reflect’ the client in a neutral and non-interfering manner.

It is also interesting to note that, contrary to strategies like repetition and contiguity (Ferrara, 1994), the present linguistic display of synchrony does not seem to be based on taking up each other’s keywords or phrases. This is consistent with the earlier observation that content words may be less revealing of interactional stances (Doyle and Frank, 2016), and will be further illustrated by the next extract where we see the converse case of high repetition but low synchrony.

Extract 2 is from Dyad B (CBT) Session 6. Recall that all sessions in dyad B are asynchronized. The therapist establishes the client’s “dire need for approval” from her mother as a key irrational belief to be disputed, and asks her to identify more potential irrational beliefs. The therapist then proceeds to point out why they are irrational.

1. THERAPIST: So this need for approval, this dire need for approval, and very pointedly from your parents, maybe more so from your mother, is going to keep you suffering if you do not continue the good work you are doing. So, any more irrational beliefs before you dispute the life out of these ones right here and now?
2. CLIENT: Yeah. Let me think.” I should have not been so impulsive to say what I was feeling at the time.”
3. THERAPIST: And because I was so impulsive, that makes me…
4. CLIENT: “And because I was so impulsive, that makes me a thoughtless daughter.”
5. THERAPIST: As I should not be.
6. CLIENT: “As I should not be.” See, it’s always that ending.
7. THERAPIST: Well it usually is that ending, if the result is anxiety, panic or other unpleasantries.
8. CLIENT: Right. God. That part always gets me.
9. THERAPIST: You mean, until now, that part has not been as evident as you’d like it to be. You cannot say it always gets you, because always implies always: past, present and future, and you still have years of life left.

As mentioned earlier, both speakers echo each other from Turns 3 to 6. The therapist guides this process by repeating key parts of the client’s utterances to prompt further reflection, and the client repeats them again (“I was so impulsive, that makes me…,” “As I should not be”) to demonstrate this reflection. Such repetitions and overlaps are expected when dysfunctional thoughts, beliefs, assumptions, etc. are discussed because they often involve concrete details depicted by content words. However, in accordance with LIWC’s non-emphasis on content words, this apparent synchrony does not translate to an actual high synchrony measure. Referring again to Table 2, the therapist and client have very different scores for all variables: *analytical thinking* (Client = 4.23, Therapist = 15.01), *clout* (Client = 38.41, Therapist = 91.05), *authenticity* (Client = 73.35, Therapist = 44.97), *emotional tone* (Client = 34.36, Therapist = 74.45), which suggest highly divergent interactional stances. The contrast in clout is evident from Turn 1 as the therapist uses many client-directed pronouns (“if you do not continue…,” “before you dispute…”) to assume an expert-like and directive stance to establish the “disput(ing) the life out of” of irrational beliefs as a key focus of their interaction. The client obliges by reflecting on the therapist’s directions using self-directed pronouns (“I was so impulsive,” “I should not be”). We see the reverse pattern for authenticity—the client’s high score is reflected in her willingness to disclose her thoughts and feelings, which is unsurprising given the present therapist-directed focus on her irrational beliefs. By contrast, the therapist’s low score is attributable to her exclusive focus on the client. The therapist’s higher scores for *analytical thinking* and *emotional tone* are likewise predictable from her general educative, problem-focused, and task-based stance. Her language contains more logical markers (“if,” “because,” “until”) compared with the client’s complementary narrative style, and she is obliged to use more optimistic or emotionally positive language in contrast with the client’s generally negative depiction of her situation. This becomes apparent later in the session (not in extract) when the therapist urges the client to remind herself that she is worth ‘approval, adoration, and acceptance’.

Our analyses of extracts 1 and 2 have shown how a quantitative contrast in synchrony measures is reflected in equally contrastive interactional stances between dyads A and B. We now consider dyad C (humanistic therapy), which has a synchrony measure (25%) midway between A and B. Extract 3 is from Session 9 where the client relates her frustrations with her mother and why she has been avoiding her. The therapist guides the client to explore these feelings and suggests that the avoidance is linked with her need to retain her sense of self.

1. CLIENT: Like I have not done a damn thing about my mother since July and she’s been through hell since then, and I just completely turned off.
2. THERAPIST: You have some feeling like there’s some resemblances between what she does and how you are sometime, that it’s absolutely necessary that you keep yourself separate from her?
3. CLIENT: To a degree yeah, mostly because I have not sorted out what’s keeping myself separate is in a way like she’s fantastically… like I’m upset in five minutes after being with her. She’s just overwhelmingly….
4. THERAPIST: In all of these.
5. CLIENT: She’s like, “I am helpless. You do not talk to me.” It’s every kind of accusation except that she never says it that way, so you cannot yell at her for saying it that way. It’s always done in a nice, rational tone.
6. THERAPIST: Man, those things can cripple.
7. CLIENT: I’ve experienced them really crippling, and I have experienced being literally nervous wrecks for a long time growing up, […] the only time that I’ve been able to handle her has been in the last year since I’ve been able to not treat her like a human being, but treat her like a patient. But if I once let myself soften to her, I am so vulnerable.
8. THERAPIST: I know. It’d almost be a bad thing tucked away or being lost.
9. CLIENT: Because I do not know who I am, but I know that I’m a very negative person from her. I cannot take that from her anymore, every time she staggers or it’s just like you just feel like crying, she’s so pitiful, and she’s also so good-hearted. I empathize with her too much, and I know what she feels like too much, and I know how she’s not able to cope, and it hurts.
10. THERAPIST: And it’s like there’s too much overlap, it not only ends up maybe damaging you, but making it really hard for you to… It may sort of feel weak or like meaningless to someone else, but like it really helps to have that, at that point because it sounds like in one way you feel the same way. You’ve got to have that separation to retain any sense of yourself.
11. CLIENT: Um hmm.
12. THERAPIST: …sense of yourself.

From Table 2, we see that the synchrony of this session is attributable to similar scores for *analytical thinking* (Client = 7.4, Therapist = 5.65) and *authenticity* (Client = 80.6, Therapist = 78.43), with larger differences in *emotional tone* (Client = 33.2, Therapist = 43.22) and especially *clout* (Client = 19, Therapist = 47.84). In this sense it lies between extract 1 where three variables are highly similar, and extract 2 where none of the variables are. Interestingly, this coincides with the fact that the overall synchrony measure of dyad C is also midway between A and B.

A closer analysis of the interactional construction of synchrony indeed reveals elements that resemble both extracts 1 and 2. The therapist attempts to clarify the client’s feelings by paraphrasing her account more precisely like ‘keep yourself separate from her’ (Turn 2) and “those things can cripple” (Turn 6). The observed concord in extract 1 is noticeable here as the client shows tacit agreement by echoing these utterances in the following turns (“what’s keeping myself separate,” “really crippling”), and markers of agreement like “(to an extent) yeah” (Turn 3) and “un hmm” (Turn 11). This general dynamic accounts for their similar *analytical thinking* and *authenticity* scores. Both score low in the former as the conversation is informal and narrative-like, and high in the latter as the client’s disclosure of her feelings (“I have not done a damn thing about my mother,” “I”m upset in 5 min, “I am so vulnerable”) is met with the therapist’s open and empathetic understanding (“Man, those things can cripple,” “I know”). Their *emotional tone* scores are not as similar, but both tend towards the negative end. Their use of negative emotion words is consistent throughout as the client relates her and her mother’s feelings (“upset,” “helpless,” “vulnerable,” “hurts”), and the therapist focuses more on the personal meaning of her experiences (“lost,” “damaging,” “weak”).

However, while the therapist in extract 1 does not seem to take the lead, the therapist here is subtly leading the process by summarizing the client’s reflections, drawing out their implications, and proposing an interpretation that is expected to be accepted (Antaki et al., 2005). This is closer to the therapist’s educative stance in extract 2 and accounts for the disparity in *clout*. Notice also that the aforementioned concord is demonstrated to a lesser extent here. The client appears to agree with the therapist (only) “to a degree” (Turn 3), and unlike extract 1, she expresses her feelings more independently and does not orient her utterance as a response to the therapist at every turn.

In summary, the above analyses attempted to contextualize the quantitative synchrony measures and illustrate how linguistic (a)synchrony can be constructed in different ways that can be examined at the individual dyadic level. Our examples generally reflect characteristics expected at the theoretical level of therapy type—dyad A demonstrates a high level of non-judgmental “reflection” often discussed in psychoanalysis, dyad B presents a sharp contrast where the CBT therapist adopts a more institutionalized educative role, and dyad C contains element of both in the humanistic therapist’s broad adoption of a guiding, empathetic approach.

## Conclusion

We demonstrated a systematic and replicable approach that combines automated quantification of therapist and client language, cluster analysis, and discourse analysis to model linguistic (a)synchrony in therapist–client interaction. While informed by existing strands of research, its use of the session as the key unit of analysis and measurement of (a)synchrony at dyadic level presents a more intuitive account than alternatives like single turns or topics. The contextual elaboration of quantitative measures affirms the importance of a mixed-method orientation to linguistic (a)synchrony research, complementing general patterns found in a dataset with a more critical eye on higher-order communicative strategies and phenomena.

Our sample analyses of three dyads from key therapy approaches were performed with both researcher and practitioner objectives in mind. The present analyses are far from representative of the therapy approaches. Depending on their interests, researchers may adopt a similar comparative approach on more representative datasets to study how (a)synchrony varies across therapy types, the temporal distribution of (a)synchronized sessions within a dyad, and/or conduct further qualitative analyses of (a)synchrony construction from different theoretical perspectives. Interested practitioners who have the required resources and skills can apply it to their own work and critically reflect on their socio-psychological stances vis-à-vis their clients, as well as their avowed therapeutic approach. It would be particularly interesting to track how one’s tendency to (a)synchronize changes across different clients and over time. Additionally, the approach could also be applied to other social contexts where there is an interest in examining linguistic (a)synchrony between speakers and across motivated intervals, such as classroom interaction (e.g., teacher vs. student talk across lessons) or online fora (different posts across time). The approach can also be attempted on quantification schemes other than LIWC that reflect different theoretical assumptions and underpinnings.

Lastly, as alluded to earlier, while it is tempting to suggest that higher linguistic synchrony measures correlate with better treatment or interactional outcomes, such questions are beyond the present scope. This is especially pertinent given that there is evidence for the general effectiveness of all three therapy types, which raises the question of how detrimental a seemingly low-synchrony approach like CBT truly is. Our approach shares with most psychotherapy language research the inherent limitations of secondary analysis of transcripts, and has a more descriptive focus on modeling rather than prescribing language use. It nevertheless provides a basis for future work to incorporate outcome measures and investigate links between linguistic synchrony and treatment quality. Relatedly, we have not considered how linguistic constructions of (a)synchrony might vary along demographic variables like age and gender, and its non-verbal manifestations like gestures and other paralinguistic cues. These can be flexibly incorporated in future work because of the schematic nature of our proposed approach. Quantification and clustering can be extended to non-linguistic or paralinguistic behaviors and variables, and the subsequent qualitative analytic phase is not married to any particular framework. The above considerations also apply to other potential contexts of linguistic (a)synchrony where our approach can be applied.

## Data Availability Statement

The original contributions presented in the study are included in the article/supplementary material, further inquiries can be directed to the corresponding author.

## Author Contributions

DT conceptualized the study including its objectives, design, methods, and analysis and also responsible for drafting the manuscript. HQ contributed to sections of the qualitative analysis. All authors contributed to the article and approved the submitted version.

## Funding

This work is supported by a Faculty Reserve Grant (ZVY8) of the Faculty of Humanities, The Hong Kong Polytechnic University.

## Conflict of Interest

The authors declare that the research was conducted in the absence of any commercial or financial relationships that could be construed as a potential conflict of interest.

## Publisher’s Note

All claims expressed in this article are solely those of the authors and do not necessarily represent those of their affiliated organizations, or those of the publisher, the editors and the reviewers. Any product that may be evaluated in this article, or claim that may be made by its manufacturer, is not guaranteed or endorsed by the publisher.

## Footnotes

^1^ https://search.alexanderstreet.com/psyc
